# Spike-Timing of Orbitofrontal Neurons Is Synchronized With Breathing

**DOI:** 10.3389/fncel.2018.00105

**Published:** 2018-04-20

**Authors:** Áron Kőszeghy, Bálint Lasztóczi, Thomas Forro, Thomas Klausberger

**Affiliations:** Division of Cognitive Neurobiology, Center for Brain Research, Medizinische Universität Wien, Vienna, Austria

**Keywords:** orbitofrontal cortex, ventral prefrontal cortex, medial prefrontal cortex, respiratory rhythm, hippocampal theta oscillations, olfactory bulb, delta oscillations

## Abstract

The orbitofrontal cortex (OFC) has been implicated in a multiplicity of complex brain functions, including representations of expected outcome properties, post-decision confidence, momentary food-reward values, complex flavors and odors. As breathing rhythm has an influence on odor processing at primary olfactory areas, we tested the hypothesis that it may also influence neuronal activity in the OFC, a prefrontal area involved also in higher order processing of odors. We recorded spike timing of orbitofrontal neurons as well as local field potentials (LFPs) in awake, head-fixed mice, together with the breathing rhythm. We observed that a large majority of orbitofrontal neurons showed robust phase-coupling to breathing during immobility and running. The phase coupling of action potentials to breathing was significantly stronger in orbitofrontal neurons compared to cells in the medial prefrontal cortex. The characteristic synchronization of orbitofrontal neurons with breathing might provide a temporal framework for multi-variable processing of olfactory, gustatory and reward-value relationships.

## Introduction

Neuronal network oscillations of different frequencies are fundamental in the flexible and quick modulation of communication between cell populations and brain areas, and thus in temporal binding, selection and integration of information (Gray et al., 1989; Buzsaki and Draguhn, 2004; Buschman and Miller, 2007; Womelsdorf et al., 2007; Klausberger and Somogyi, 2008; Lapray et al., 2012). The respiratory rhythm has a strong influence on olfactory processing, and it is integral to olfactory perception (Macrides and Chorover, 1972; Kepecs et al., 2007; Verhagen et al., 2007; Shusterman et al., 2011; Fukunaga et al., 2012); accordingly, local field potential (LFP) oscillations coherent with breathing have been documented in olfaction related and other brain regions, including the olfactory bulb (OB; Adrian, 1942, 1950; Rojas-Libano et al., 2014), the piriform cortex (Fontanini et al., 2003), the barrel cortex (Ito et al., 2014), the hippocampus (HIP; Macrides et al., 1982; Vanderwolf, 1992; Yanovsky et al., 2014; Lockmann et al., 2016; Nguyen Chi et al., 2016) and more recently in the medial prefrontal cortex (mPFC; Biskamp et al., 2017; Zhong et al., 2017; Tort et al., 2018). As a prefrontal area the orbitofrontal cortex (OFC) is not regarded as a primary olfactory structure but contains odor responsive neurons (Rolls and Baylis, 1994), and receives substantial inputs from olfactory areas (Price, 1985; Chen et al., 2014), posterior domains of it are considered to incorporate secondary and tertiary olfactory cortices and representations of odor identity and valance (Rolls, 2004). Among many other functions OFC is also implicated in representation of complex flavors (by integration of primary taste and olfactory information), subjective momentary food reward value, and different properties of expected outcomes (Rolls, 1989, 2015; Rolls and Baylis, 1994; Gallagher et al., 1999; Rudebeck and Murray, 2011; Wallis, 2011; Noonan et al., 2012; Lak et al., 2014; Lopatina et al., 2015; Stalnaker et al., 2015). Phase coupling of OFC units to a 4–12 Hz LFP oscillation has been reported during odor sampling and reward expectation (van Wingerden et al., 2010a,b), but has not been linked to respiration. This frequency range could be related to both hippocampal theta oscillations (5–12 Hz) and the breathing frequency, which varies considerably with behavior, e.g., locomotion and immobility (2–12 Hz in rodents; Welker, 1964; Kepecs et al., 2007; Nguyen Chi et al., 2016). To facilitate understanding of the complex information processing in the OFC, we investigated whether OFC LFP oscillations and the breathing are coherent at different respiratory frequencies, and if so whether the firing of OFC neurons is modulated by these rhythms.

## Materials and Methods

### Animal Care and Handling

For this study, 10 adult, male, C57 BL6 mice were used. This study was carried out in accordance with the recommendations and approved licence of the Austrian Ministry of Science and the Medical University of Vienna. The protocol was approved by the Austrian Ministry of Science and the Medical University of Vienna. Animals were implanted with a custom-made head-plate (under isoflurane anesthesia), to allow for head-fixation, and electrophysiological recordings. After at least 4 days of recovery from the implantation surgery, water restriction of the animals was initiated, to facilitate handling and acclimatization to head-fixation and the virtual environment (Jet-Ball, Phenosys, Berlin, Germany). This procedure was similar to what had been suggested earlier for head-fixed experiments (Guo et al., 2014), mice were given 1–1.3 ml water every day, and among other parameters their body weight was monitored and kept 80%–90% of their original mass. The running periods of the animals were identified based on the recorded X-Y coordinates of the jet-ball and speed in the virtual environment. After animals were comfortable with the head-fixation and learnt to move in the virtual environment, a craniotomy and duratomy was performed (under isoflurane anesthesia), to allow for silicon probe penetration and electrophysiological recordings from the OFC and mPFC.

### Breathing Monitoring

**Figure 1:**
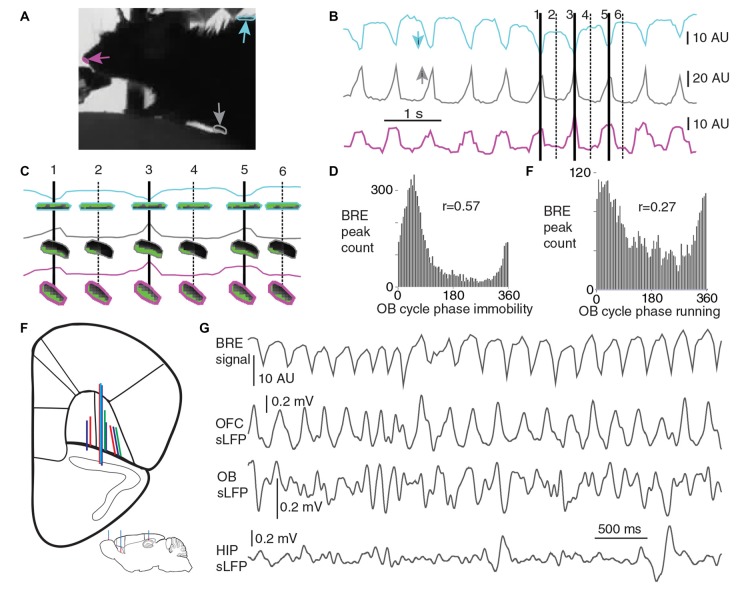
Breathing monitoring and electrophysiological recordings. **(A)** Breathing was monitored in the head-fixed animals utilizing a video-based method. Three region of interests (ROIs) are highlighted at the back, abdominal and nose-contour regions of the animal. **(B)** The average light intensity values within these ROIs are plotted as a function of time. During immobile periods, breathing cycles result in small back and forth displacement of the contour within all of these ROIs, which result in the fluctuation of average light intensity calculated from these regions. Small arrows color-matched with the respective breathing signal traces are indicating the direction toward inhalation-climax times. For three consecutive cycles inhalation-climax times are highlighted with solid lines and numbers, the time-matched snapshots of the ROIs are shown enlarged in **(C)**, pixels with higher value than a threshold are shown in green. During immobility, inhalation-climax times were detected based on the signal from the abdominal or back ROIs, whereas during running the nose ROI signal was used only. **(D,E)** Strong phase coupling of the detected breathing peaks to local field potential (LFP) cycles in the olfactory bulb (OB) was found during immobility (**D**, *p* < 0.0001, Rayleigh test, n: 7744 cycles) and running periods (**E**, *p* = 3.28 × 10^−133^, Rayleigh test, n: 4258 cycles), confirming the validity of the breathing detection. **(F)** Inset, experimental design on a para-sagittal scheme of the mouse brain. Multi-site silicon probe recordings were carried out in the orbitofrontal cortex (OFC), while breathing rhythm, hippocampal and OB LFP was recorded simultaneously. The coronal outline summarizes the tracks of silicon probes from 10 recording sessions from four mice (matching colors indicate the same animal). **(G)** Recording trace with breathing signal and LFP from OFC, OB and hippocampus (HIP; immobile period); note the preferential occurrence of OFC LFP peaks in time windows centered around the inhalation peaks.

Breathing rhythm was monitored with a video-based method, based on average pixel intensity changes in regions of interest (ROI) from the abdominal, back and nose contour regions of the head-fixed mice. A 25 Hz frame rate video, synchronized with the electrophysiological recording, was captured from the animals (with the Spike 2 Video software), with a side point of view; the monitors of the virtual environment created a back lit situation, resulting in a high contrast contour of the animal. When the animals were sitting or standing still (immobility), breathing-related movements were visible on the video, resulting in coherent fluctuations in the average pixel intensities within all three ROIs (Figures 1B,C), with a higher signal to noise ratio at the abdominal and back ROIs as opposed to the nose ROI, therefore during immobile periods the earlier two ROIs were utilized for the breathing monitoring. The nose ROI signal was only exerted for the running periods, when the signal from the other two ROIs was not usable for breathing monitoring. During running periods the abdominal and back ROI signal fluctuations were reflecting the locomotion related movements and not the breathing-related movements. The head-fixation ensured that the skull was still during the running periods also, allowing for the utilization of the nose ROI signal. Mean pixel intensity fluctuations were calculated for each ROI in FIJI ImageJ software. Breathing cycle borders were defined by inhalation-climax times, based on detected troughs from the back ROI, or peaks from the abdominal or nose-ROIs (both of these event times were automatically detected in the Spike 2 software with a threshold based method, in the identified and visually validated immobility and running periods where the signal to noise ratio was high enough). The detected inhalation-climaxes were strongly phase coupled to slow OB LFP cycles during both immobility and running periods (Figures 1D,E), this can be considered as an indirect validation of our breathing monitoring method, as it had been described that the slow OB LFP cycles follow the breathing rhythm in a cycle by cycle manner (Rojas-Libano et al., 2014).

### Electrophysiological Recording

For the silicon probe experiments, we recorded OFC LFP and spiking activity from four animals (10 recording sessions in total), the OB LFP was recorded from all of these animals (all 10 recording sessions), and the HIP LFP was recorded from two out of these four animals (seven recording sessions). mPFC spiking activity and LFP was recorded from two animals (two recording sessions). From one animal both OFC and mPFC recordings were made. Spiking activity and LFP was recorded with 16 and 32 channel silicon probes (SP, NeuroNexus, Ann Arbor, MI, USA), from the prefrontal cortex, in acute experiments, 30 min after probe penetration (linear 16 and poly3 32 site layout probes were used for the OFC recordings, four shank tetrode and four shank linear probe layouts were used for the mPFC recordings). Silicon probe track locations were post-fix analyzed and validated. Based on known shank or penetration distances, a scaling factor was determined for each brain separately, site locations were mapped on the coronal slices afterwards according to this scaling, using the SP tip location as a reference point. The orbitofrontal silicon probe penetrations were ranging from 2.1 mm to 2.46 mm anterior and 1.02 mm to 1.6 mm lateral to Bregma, covering the lateral part of ventral-OFC and the lateral-OFC, in the right hemisphere (Figure 1F); mPFC recording sites were located in prelimbic and infralimbic cortex (right hemisphere). Potentials were recorded with a 20 KHz sampling rate (with a CED multichannel AD converter, with Spike 2 software) after 10 KHz low-pass filtering and a 1000 times amplification (with Neura Lynx amplifiers and Tucker and Davis pre-amplifiers). Hippocampal LFP was recorded through a teflon coated wire, implanted 2.3 mm caudal and 1.5 mm lateral to Bregma, the penetration was 1.05 mm deep from the brain surface, resulting in a target location which is dorsal from the pyramidal layer in the dorsal HIP (right hemisphere, Figure 1F inset). OB LFP (surface ECoG) was recorded through a screw traversing the skull but not the dura mater above the right OB (4.3 mm anterior, 0.8 mm lateral to Bregma, Figure 1F inset). Juxtacellular recording was performed in five animals as described earlier (Lapray et al., 2012), in short 12–30 MΩ glass electrodes, filled with 3% neurobiotin (Vector Laboratories) in 0.5 M NaCl solution were used. Electrophysiological signal was amplified 1000-times with an NPI ELC 100 M headstage and NPI amplifier (BF-48DGX, DPA-2FS and ELC-01MX, NPI Electronic GmBH), and sampled at 20 KHz. After extracellular recording of spiking activity of OFC cells, juxtacellular labeling was performed as described earlier (Klausberger et al., 2003).

### Data Analysis

**Figure 2:**
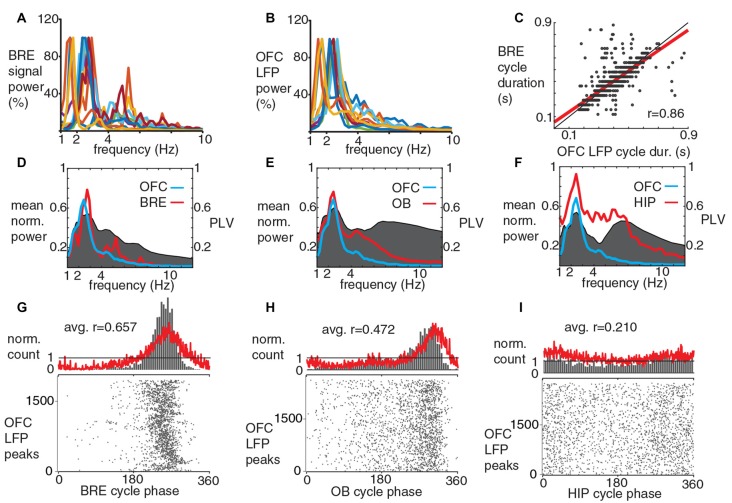
The dominant slow LFP oscillatory component in mouse OFC is synchronous with breathing during immobile periods. **(A,B)** Power spectra for breathing signal and OFC LFP, different colors indicate individual recording sessions. Note the similar peak frequencies for the averaged power spectrums **(D)**. **(C)** OFC LFP and breathing cycles show a strong cycle by cycle correlation in their durations, the least squares fit line is shown in red (one recording session, 1938 cycles, Spearman’s correlation coefficient: 0.86). **(D–F)** Coherence (gray, right y-axis) and averaged power spectra (red and blue left y-axis) of and between breathing and LFPs in OFC, OB and HIP. PLV, phase locking values. **(G–I)** Phase-coupling histograms for OFC LFP peaks to breathing cycles (**G**, 10 recording sessions, four animals), to OB LFP cycles (**H**, 10 recording sessions, four animals) and to HIP LFP cycles (**I**, seven recording sessions, two animals). Red curve, averaged phase histogram for all recording sessions, gray raster and column charts show data from a single recording session. OFC LFP peaks were coupled significantly stronger to breathing cycles compared to OB LFP cycles (*p* = 0.00389; Mann-Whitney test, after Bonferroni correction alpha = 0.0167), and compared to HIP LFP cycles (*p* = 0.0001; Mann-Whitney test, after Bonferroni correction alpha = 0.0167). Gray columns, normalized cycle count per bin. Bottom, gray raster plots show cycle-by-cycle data during a single recording session.

In this study, we only analyzed slow oscillatory components of the LFP, after a 1–12 Hz band pass filtering (when we refer to LFP, this frequency band is considered). OFC LFP was always taken from a silicon probe site located in cortical layer 2. OFC and HIP LFP oscillatory cycles were defined by peaks, OB LFP cycles were defined by troughs (both of these event types were detected automatically by the Spike 2 software with a threshold based method). To characterize LFP oscillations power spectrums were generated in the Spike 2 software for each recording session separately (Figures 2A,B), average power spectrums were generated in Matlab, after normalization for the highest peak in the 1–12 Hz frequency range (Figures 2D–F). To assess coherence between different LFPs and the breathing rhythm coherence spectrograms were generated in Matlab (Figures 2D–F, 5A–C), from phase locking values (PLV; Lachaux et al., 1999), calculated with 0.11 Hz frequency resolution, after wavelet transformation (a complex Morlet wavelet transform was used in the 1–12 Hz frequency range, to produce 100 logarithmically equidistant frequencies components, wavelet parameters of 1 and 1.8 were used, Matlab Wavelet Toolbox). To characterize the phase coupling phenomena between different LFPs and the breathing rhythm average phase histograms and average r-values (ArV; average of the mean vector lengths) were calculated (Figures 2G–I, 5D–F, red curves); from the individual phase histograms and r-values determined for each recording session separately (individual phase histograms were generated in the Spike 2 software; r-values were calculated in Matlab, utilizing the circular statistics toolbox; Berens, 2009). For the statistical comparison of coupling strength of breathing to different LFP components, Bonferroni corrected Mann-Whitney test was used (Matlab Statistics Toolbox).

**Figure 3:**
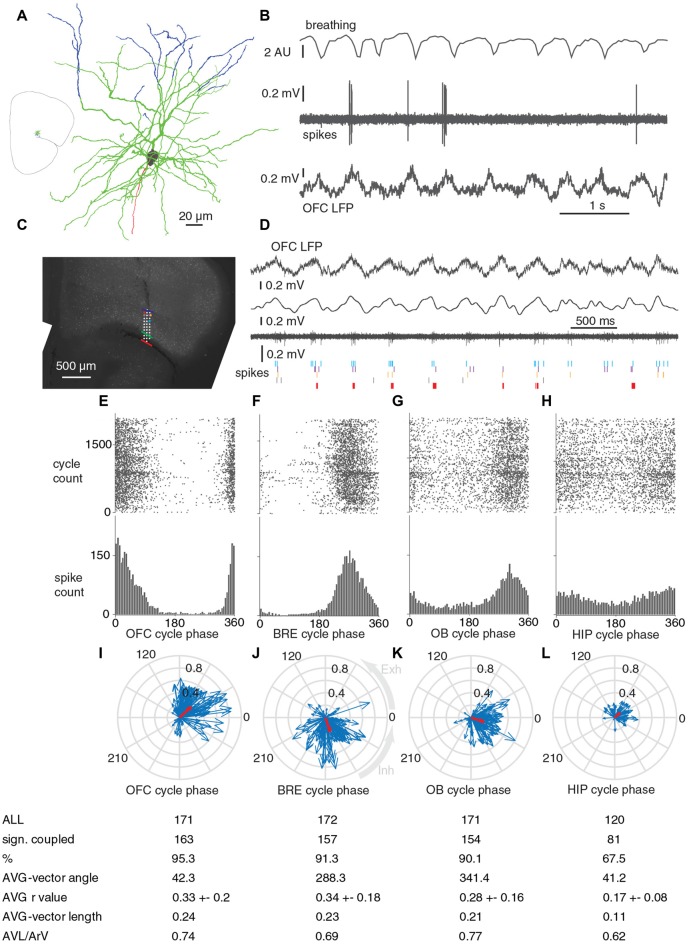
Units in OFC fire phase-coupled to breathing and breathing-synchronous LFP oscillations during immobility. **(A,B)** Reconstruction and firing of a recorded and juxtacellulary labeled pyramidal cell in the layer 2 of OFC. **(A)** Blue, dendrites in layer 1; green, dendrites in layer 2/3; gray, soma; red, axon. Inset, orientation of the recorded and labeled cell in a brain section. **(B)** The labeled neuron fires phase-coupled to breathing and slow oscillations in the LFP. **(C)** Sites of a poly-3 silicon probe projected on the respective coronal brain section with immunohistochemical labeling for parvalbumin. Red, green and blue lines represent boarders between Piriform Cortex-OFC, OFC layer 1 – OFC Layer 2/3 and OFC layer 2/3 – OFC layer 5, respectively. **(D)** From top to bottom: wideband LFP in OFC layer 2/3, filtered (0.1–12 Hz) LFP in OFC layer 2/3, multi unit activity in OFC layer 2/3, and spike timing of five OFC neurons detected from the same and neighboring sites (their locations are indicated with matching colored dots in **(C)**. **(E–H)** Phase coupling of the spikes of an OFC unit (shown with light blue on **B**) to OFC LFP, breathing, OB LFP and HIP LFP cycles, respectively. **(I–L)** Circular phase plots of all the significantly coupled OFC units to OFC LFP, breathing, OB LFP and HIP LFP cycles, respectively. Each blue arrow represents a significantly coupled unit, the arrow-angle shows the mean phase of coupling, whereas the arrow-length shows the strength of the coupling. Red arrows show the average vector. Average coupling strength was highest for BRE cycles, it was not significantly lower for OFC LFP cycles (*p* = 0.695, Mann-Whitney test, n: all significantly coupled clusters, indicated on **I,J**, after Bonferroni correction alpha = 0.0083), but it was significantly lower for OB LFP cycles (*p* = 0.0064, Mann-Whitney test, n: **J,K**, after Bonferroni correction alpha = 0.0083) and HIP LFP cycles (*p* = 4.5 × 10^−13^, Mann-Whitney test, n: **J,L**, after Bonferroni correction alpha = 0.0083). Abbreviations: AVG, average; AVL, average-vector-lengths; ArV, average r-values.

**Figure 4:**
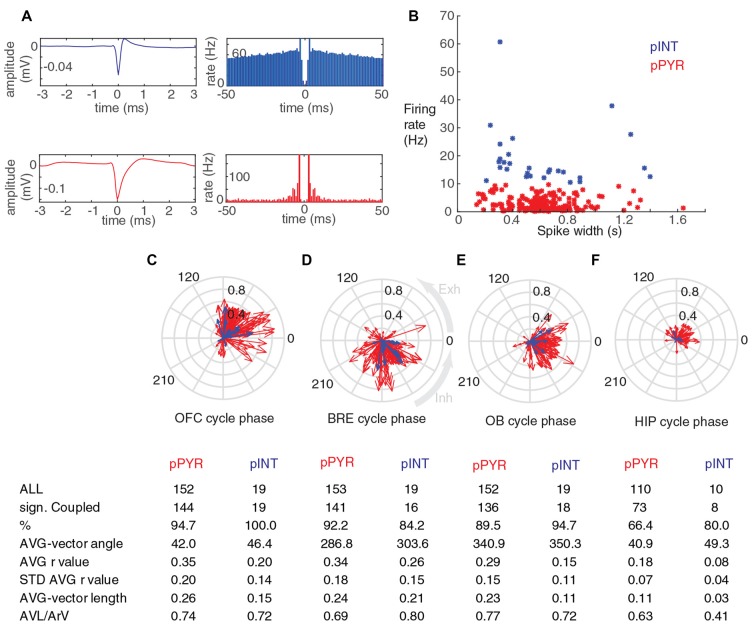
Putative OFC pyramidal cells and interneurons phase-couple to breathing during immobility. **(A)** Average spike shapes and autocorrelograms of a putative interneuron and a putative pyramidal cell from the OFC (top and bottom row respectively). **(B)** Scatter plot showing over all firing rate and spike width for all silicon probe recorded units. Putative pyramidal cells and putative interneurons were assigned to these categories based on a 10 Hz firing rate threshold, and shown in red and blue respectively. **(C–F)** Circular phase plots of all the significantly coupled OFC units to OFC LFP, breathing, OB LFP and HIP LFP cycles, respectively. Each red arrow represents a significantly coupled putative pyramidal cell, each blue arrow represents a significantly coupled putative interneuron, the arrow-angle shows the mean phase of coupling, whereas the arrow-length shows the strength of the coupling. The average vector angle and AVL/ArV ratios for putative OFC interneurons and putative pyramidal cells indicated that these categories have similar phase preference and phase preference consistency on the population level **(C–F)**. Average coupling strength of putative pyramidal neurons to breathing was high, and it was not significantly different from the average coupling strength of these cells to OFC LFP and OB LFP cycles (*p* = 0.96 and *p* = 0.036 respectively, Mann-Whitney test, n: all significantly coupled clusters, indicated on **(C–E)**, after Bonferroni correction alpha = 0.0083), but the average coupling strength of these cells was significantly lower to HIP LFP cycles (*p* = 1.1 × 10^−11^, Mann-Whitney test, n: **(D,F)**, after Bonferroni correction alpha = 0.0083). Average coupling strength of putative interneurons to breathing was high, and it was not significantly different from the average coupling strength of these cells to OFC LFP and OB LFP cycles (*p* = 0.2 and *p* = 0.02 respectively, Mann-Whitney test, n: all significantly coupled clusters, indicated on **(C–E)**, after Bonferroni correction alpha = 0.0083), but the average coupling strength of these cells was significantly lower to HIP LFP cycles (*p* = 0.004, Mann-Whitney test, n: **D,F**, after Bonferroni correction alpha = 0.0083).

**Figure 5:**
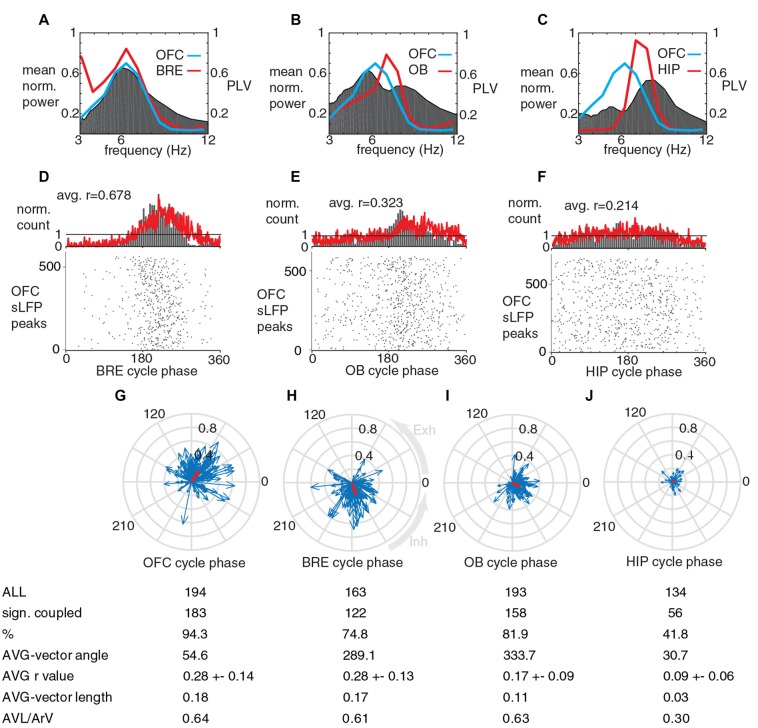
During running periods, the neurons in OFC fire phase-coupled to a breathing-coherent slow LFP oscillatory component. **(A–C)** Coherence (gray) and averaged power spectra (red and blue) of and between breathing and LFPs in OFC, OB and HIP during running periods; note matching peak frequencies. **(D,E)** Phase-coupling histograms for OFC LFP peaks to breathing cycles (**D**, 10 recording sessions, four animals), to OB LFP cycles (**E**, nine recording sessions, four animals) and to HIP LFP cycles (**F**, seven recording sessions, two animals). Red curve, averaged phase histogram for all recording sessions. Gray columns, normalized cycle count per bin for a single recording session. Bottom, gray raster plots show cycle-by-cycle data during a single recording session. OFC LFP peaks were coupled significantly stronger to breathing cycles compared to OB LFP cycles (*p* = 2 × 10^−5^; Mann-Whitney test, after Bonferroni correction alpha = 0.0167), and compared to HIP LFP cycles (*p* = 0.0001; Mann-Whitney test, after Bonferroni correction alpha = 0.0167). **(G–J)** Circular phase plots of significantly coupled OFC units to OFC LFP, breathing, OB LFP and HIP LFP cycles during running periods. Average coupling strength was highest for BRE cycles, it was not significantly lower for OFC LFP cycles (*p* = 0.584, Mann-Whitney test, n: all significantly coupled clusters, indicated on **(G,H)**, after Bonferroni correction alpha = 0.0083), but it was significantly lower for OB LFP cycles (*p* = 9 × 10^−14^, Mann-Whitney test, n: **H,I**, after Bonferroni correction alpha = 0.0083) and HIP LFP cycles (*p* < 1 × 10^−15^, Mann-Whitney test, n: **H,J**, after Bonferroni correction alpha = 0.0083).

Unit spiking activity was assessed by automatic clustering with the python based Klusta software (Rossant et al., 2016), followed by visual validation of clusters. Units which fired less than 50 spikes during the included running or immobility periods, were excluded from the analysis. Putative pyramidal cells and putative interneurons were differentiated from each other by an arbitrary 10 Hz overall firing rate threshold (Figures 4A,B; Malagon-Vina et al., 2018), this method cannot guarantee perfect pyramidal cell vs. interneuron separation, but ensures an enrichment of interneurons in the high firing rate category and the enrichment of pyramidal cells in the low firing rate group. In addition, we obtained similar separation of units with a K-means clustering algorithm, utilizing three parameters: spike width; spike symmetry; and overall firing rate. Spike width is defined at 10% of the spike amplitude, spike symmetry is defined by the difference between 10%-rise-time and 10%-decay-time divided by the spike width. Running and immobility periods were identified based on the position and speed information from the virtual environment, and only those periods were kept where the breathing was detectable. For each cluster, the significance of coupling (to all three LFPs and to the breathing rhythm, Rayleigh test, Matlab, circular statistics toolbox; Berens, 2009), the mean phase of coupling, and the r values (mean vector length) were determined. To provide a measure of the reliability of these methods we calculated the r values before and after shuffling of spike times (1 recording session, 44 significantly coupled units; for each cell separately all the spike-times were shifted with a random duration within ±800 ms; 100 repetitions of shuffling). R values based on original spike times were always above than the mean of shuffled r values, and in 95.5% of the cases they were above the mean plus two STD line. Afterwards the summation of the vectors for all significantly coupled clusters was performed, where each vector was defined by the mean angle of coupling and the r-value for the given cluster, this resulted in an average-vector; next the average-vector-lengths (AVL), the average-vector-angles and the ArV, were determined, to characterize the population level coupling to each of the four oscillatory occurrences. As a simple measure of how consistent was the phase preference of units on the population level, the ratios of AVL and the ArV were calculated. The distinction of coupling strength of the same unit population to different oscillations were assessed by comparing the r value populations with the Mann Whitney Test (Microsoft Excel, and Real Statistics Resource Pack software (Release 4.9). Copyright (2013–2018) Charles Zaiontz^1^). When r-values were statistically compared between different cell populations, from different areas they were recalculated, based on including only randomly selected 50 APs from each cluster (from the immobility periods), to avoid spike number dependent r-value biases (but only for the comparisons); r-values shown in the descriptive part were calculated based on all spikes for the given cluster, for the sake of maximal possible precision.

All original data from this study will be made available upon reasonable request.

## Results

### Orbitofrontal Slow Oscillations in the LFP Are Coherent With Breathing During Awake Immobility

We performed simultaneous electrophysiological recordings of LFPs and neuronal activity in the OFC, HIP and OB together with video-based monitoring of breathing in head-fixed, awake mice. During immobility, breathing-related movements were detected at the back, the abdomen and the nose-contour of the animals, and were quantified by average pixel intensity changes in appropriately placed ROIs (Figures 1A–C and “Materials and Methods” section). Breathing cycles were defined by inhalation-climax times (taken as 0°). The accuracy of this video-based detection of breathing cycles was confirmed by our observation, that detected breathing cycles were highly coherent to slow oscillations detected in the LFP of the OB (Figures 1D,E, Supplementary Figure S1), as a strong coherence between these two rhythms had been described earlier (Rojas-Libano et al., 2014).

Silicon probes were positioned in the lateral and ventral OFC (Figure 1F). Peaks of slow oscillations in the orbitofrontal LFP occurred preferentially around inhalation peaks of breathing and simultaneous with troughs in the LFP of the OB (Figure 1G). During sitting periods, peaks in the LFP power spectra from individual OFC recording sessions ranged from 1.47 Hz to 2.69 Hz, similar to the breathing rhythm of the same periods (2.3 ± 0.5 Hz; Figures 2A,B). Spontaneous changes in breathing cycle length were linked instantaneously with similar changes in the LFP cycles of the OFC (Figure 2C). We observed that not only the dominant frequencies of OFC LFP and breathing were very similar, but a strong coherence between these oscillatory phenomena occurred at a matching frequency band, indicated by highest PLV in this range (Figure 2D). A strong coherence between OFC LFP oscillations and OB LFP oscillations (Figure 2E), as well as between OFC LFP and hippocampal LFP (Figure 2F) in the slow frequency range was observed. Furthermore, peaks in the OFC LFP cycles were phase-coupled to breathing cycles with a high precision (Figure 2G). OFC LFP oscillatory cycles were also significantly coupled to OB and HIP LFP cycles, with decreasing ArV in this order (Figures 2H,I). The phase coupling of OFC LFP peaks to breathing cycles was significantly stronger than their coupling to OB LFP and HIP LFP cycles. A slow oscillatory component of the medial prefrontal LFP was also significantly coupled to breathing (Supplementary Figure S2), as it had been described earlier (Biskamp et al., 2017; Zhong et al., 2017).

### Neurons in OFC Fire Phase-Coupled to Breathing During Immobility

As the phase-coupling of OFC LFP cycles to breathing was compelling, we tested whether this breathing-coherent rhythmicity manifests on the level of neuronal action potentials in the OFC. We extracellularly recorded the firing activity of neurons in the OFC with glass electrodes followed by juxtacellular labeling with neurobiotin for *post hoc* histological verification (*n* = 5; Figures 3A,B). Furthermore we recorded 195 OFC neurons in four animals with silicon probes. The location of the silicon probes and recording sites were verified (Figures 3C,D). We observed that more than 90% of units recorded with silicon probes and all five juxtacellularly recorded cells were significantly phase-coupled to the breathing rhythm of the animal, as well as to slow oscillations in the LFP of OFC and OB (Figures 3E–K). 67.5% of OFC neurons were phased-coupled to HIP LFP cycles (Figure 3L). On the population level, the average unit coupling strength was highest for breathing and OFC LFP cycles with an average *r* value of 0.33, and it was significantly lower for OB LFP and HIP LFP cycles, reflected by the lower ArV and average vector lengths (Figures 3I–L). Based on the average vector angle, most OFC clusters had the highest firing probability at early descending phase of the OFC LFP cycles (42 degrees after the peaks, Figure 3I), 72 degrees earlier than the inhalation-climax (Figure 3J), and 19 degrees earlier than OB LFP troughs (Figure 3K).

### Putative Pyramidal Cells and Putative Interneurons of the Orbitofrontal Cortex Phase-Couple to Breathing During Immobility

To explore potential cell type specific differences in the breathing coupling phenomenon, orbitofrontal units were assigned to putative pyramidal cell and putative interneuron categories based on an arbitrary 10 Hz overall firing rate threshold (Figures 4A,B, see also “Materials and Methods” section and Malagon-Vina et al., 2018). The average vector angle and AVL/ArV ratios for putative OFC interneurons and putative pyramidal cells indicated that these categories have similar phase preference and phase preference consistency on the population level (Figures 4C–F). During sitting periods 90% or more of putative OFC pyramidal cells were significantly phase-coupled to the breathing rhythm of the animal, and or slow oscillations in the LFP of OFC and OB (Figures 4C–E); 64.4% of putative pyramidal cells were phased-coupled to HIP LFP cycles (Figure 4F). During immobility 100% of putative OFC interneurons were significantly phase-coupled to slow oscillations in the LFP of OFC, 84.2% were coupled to breathing rhythm, 94.7% were coupled to OB LFP cycles, 80% were coupled to HIP LFP cycles (Figures 4C–F).

### Neurons in OFC Fire Phase-Coupled to the Breathing Rhythm During Running Periods

The breathing frequency was higher when the head-fixed animals were running on the jet-ball (5.4 ± 1.3 Hz; Figure 5A), as opposed to immobility (2.3 ± 0.5 Hz; Figures 2A,D). Accordingly, the dominant component of the OFC slow LFP oscillations followed this frequency increase, resulting in a preserved strong coherence between these signals (Figure 5A). During running the coherence between OFC LFP oscillations and OB LFP oscillations was high in the breathing frequency range (Figure 5B), whereas the coherence between OFC LFP and HIP LFP in the breathing frequency range was low (Figure 5C). During running, OFC LFP cycles were also significantly phase-coupled to breathing cycles, OB LFP cycles and HIP LFP cycles, with decreasing strength in this order (Figures 5D–F respectively). The phase-coupling of peaks in the OFC LFP to breathing cycles was significantly stronger than their coupling to OB LFP and HIP LFP cycles. During running, a high proportion of OFC units were significantly coupled to OFC LFP cycles, breathing cycles and OB LFP cycles; whereas the proportion of significantly coupled OFC units to HIP LFP cycles was decreased together with the average vector length and population level phase accuracy (Figures 5G–J). Average OFC unit coupling strength was highest for breathing and OFC LFP cycles, and it was significantly lower for OB LFP and HIP LFP cycles.

### The Firing of Neurons in OFC Is Stronger Coupled to Breathing Compared to Firing of Neurons in the Medial Prefrontal Cortex

**Figure 6:**
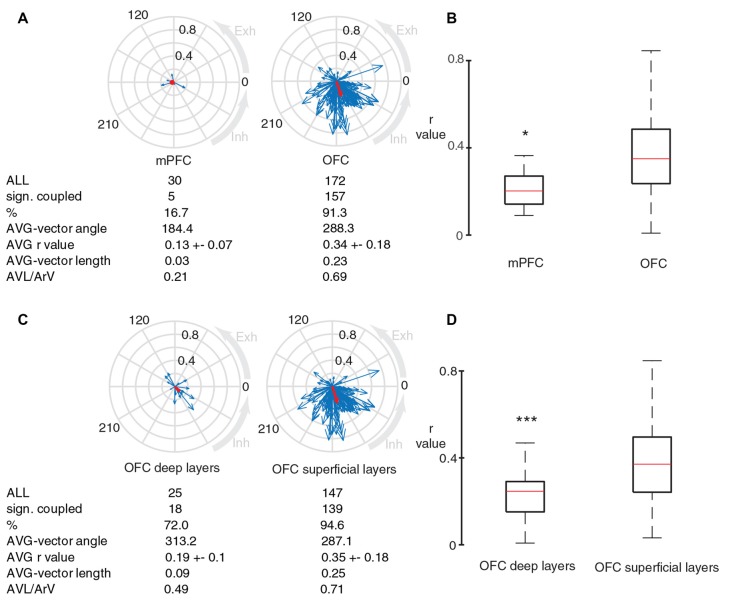
Neurons in OFC are better coupled to breathing compared to neurons in medial prefrontal cortex. **(A)** Blue arrows indicate phase-coupling strength and preferred angle of individual units from medial prefrontal cortex (mPFC) and OFC (left and right respectively, only immobile periods). Red arrows show the average vector. **(B)** OFC units have significantly stronger coupling to breathing compared to units in mPFC (*p = 0.043; Mann-Whitney test, for the comparison r-values were calculated based on randomly selected 50 APs for each cluster). **(C)** Circular plots showing the coupling strength and preferred phase of OFC neurons in deep and superficial layers to breathing cycles during immobile periods. **(D)** Box plots comparing the significantly different r-value distributions of superficial and deep OFC units (***p = 4.35e-04, Mann-Whitney test, for the comparison r-values were calculated based on randomly selected 50 APs for each cluster).

Because of our observed and surprisingly strong (*r* = 0.33) coupling of OFC neurons to breathing, we investigated if neurons in other prefrontal areas exhibit a similar link with breathing. For this, we recorded 30 neurons in the medial prefrontal cortex and observed that the percentage of units with significantly coupled firing to breathing, as well as their respective strength of coupling was significantly lower in the mPFC compared to neurons in the OFC (Figures 6A,B). This suggests a preeminent coupling of neuronal activity in the OFC to breathing.

Furthermore, we compared OFC neurons in superficial (1–3) and deep (5–6) cortical layers. The percentage of significantly coupled units was lower in deep layers (Figure 6C). The r-values from the superficial OFC layers were significantly higher than the r-values from the deep OFC layers (Figure 6D), suggesting a stronger coupling to breathing in neurons of superficial OFC layers.

## Discussion

Our data show that the firing rate of OFC neurons is strongly modulated by a breathing rhythm-coherent LFP oscillation. This coupling phenomenon was stronger in OFC as opposed to the medial prefrontal set of units. In other words mPFC and OFC cell populations relate to the same rhythm differently, which might be a reflection of different function of this oscillation in these cortical regions. There was also a coupling strength difference between superficial and deep orbitofrontal layers, the phase-coupling to breathing was the most prominent in superficial layers.

Among a multitude of functions, OFC has been suggested to execute a multi variable, subjective, momentary, food-reward-value processing function (Rolls et al., 1989; Gallagher et al., 1999; Rolls, 2000; Schultz et al., 2000; Passingham and Wise, 2012); complex flavors can be part of this equation. Integration of primary taste and primary olfactory information in the OFC is well documented by a series of studies from Rolls and colleagues (Rolls, 1989, 2015; Rolls and Baylis, 1994). Our results may have implications in this complex processing, through providing a temporal framework for integrations of olfactory and gustatory information. OFC also represent odor identities and reward values of odors (independent of taste information, Rolls, 2004), it is reciprocally interconnected with the piriform cortex (Datiche and Cattarelli, 1996; Chen et al., 2014), coupling of OFC units to breathing rhythm can be important in this reciprocal communication between these cortical areas. OFC is also supposed to reflect a high order re-representation of bodily states originating from viscero-sensory information (Craig, 2002), possibly adjusting valuation to current needs; breathing rhythm can also be considered as another dimension of this multi variable map of homeostatic state. Anxiety and mood disorders, including depression, are related to OFC (Drevets, 2007; Milad and Rauch, 2007), to breathing (Brown and Gerbarg, 2005; Kunik et al., 2005) and olfaction (Song and Leonard, 2005; Negoias et al., 2010) separately; our results can provide a link between those. OFC is also involved in the learning and reversal of stimulus outcome associations (Chudasama and Robbins, 2003; O’Doherty et al., 2003; Schoenbaum et al., 2003; Rolls, 2004; Bissonette et al., 2008), and plays a role in expectations, predictions, and representations of different properties of expected outcomes (Schoenbaum et al., 1998; Lopatina et al., 2015). OFC neurons show increased coupling to 4–12 Hz oscillations during reward expectation and odor sampling periods (van Wingerden et al., 2010a,b; Pennartz et al., 2011). Based on our data it is possible that the 4–12 Hz coupling observed by van Wingerden and colleagues reflects coupling of OFC neurons to breathing rhythms.

The relatively modest phase-coupling strength of mPFC neurons to breathing, and no strong phase preference on the population level in our experiments was similar to what was described in recent studies focusing on the mPFC (Biskamp et al., 2017; Zhong et al., 2017); the characteristic breathing frequency was different in the study from (Biskamp et al., 2017; close to 4 Hz) during potentially fearful, immobile, tail suspension periods. Fear expression-related 4 Hz oscillations have been reported to modulate neuronal activity in the mPFC (Karalis et al., 2016). Based on these findings it is possible that during fearful immobility, breathing frequency stabilizes at 4 Hz in mice, and mPFC units couple to this rhythm; see also the somatic marker hypothesis of emotions (Damasio, 1996). Increased mPFC unit coupling to a 4 Hz LFP oscillatory component has also been reported during working memory (Fujisawa and Buzsaki, 2011). This makes it less likely that the 4 Hz frequency band in rodent prefrontal cortex is preserved exclusively for fearful periods, but respiratory rhythm might still provide a common explanation, as it can have matching frequency during different behavioral conditions.

The video-based breathing monitoring technique utilized in this study, is non-invasive, as opposed to thermocouple probe implantation into the nasal cavity (Kepecs et al., 2007), or EMG from diaphragm (Rojas-Libano et al., 2014). However it has limitations as well, as short double breathes might remain undetected. Also, it requires that some parts of the animals’ body-contour (thoracic, abdominal, nostril) is free of gross movements. This makes it feasible to use in head-fixed experiments, with better performance during sitting periods. In this study visual control was performed to exclude time periods of sudden movement (e.g., grooming, scratching). This method is capable of defining breathing cycles, by inhalation-climax times, but it is not ideally suited for the precise detection of borders between all sub-cycle epochs. During running periods in the head-fixed animals only the signal from the nostril ROI can be used, which results in less sensitivity and precision of breathing cycle-border detection. During some periods of recording, the breathing was not detectable, resulting in running periods which are not included in our analysis. Camera positioning, contrast between background and animal, selection of ROIs do effect the sensitivity of the method, especially in the case of the nostril ROI and running periods.

In summary, firing of neurons in the OFC exhibit a strong temporal relationship with the breathing rhythm, which might provide a temporal framework for high order, multi-dimensional processing of sensory, value and internal state information.

## Author Contributions

ÁK, BL and TK contributed to the conception and design of the study and wrote the manuscript. ÁK carried out the silicon probe experiments and performed the analysis of the data. ÁK, BL and TF carried out the juxtacellular recording and labeling experiments.

## Conflict of Interest Statement

The authors declare that the research was conducted in the absence of any commercial or financial relationships that could be construed as a potential conflict of interest.

## Footnotes

^1^www.real-statistics.com
