# Supplementary material for: Spike-Timing of Orbitofrontal Neurons Is Synchronized With Breathing
Source: Front Cell Neurosci. 2018 Apr 20;12:105. doi: 10.3389/fncel.2018.00105 (PMC5920025; doi:10.3389/fncel.2018.00105)
Supplement: Supplementary file 1 [file Presentation_1.PDF]

Supplementary figures

Spike-timing of orbitofrontal neurons is synchronized with breathing

Áron Kőszeghy[1], Bálint Lasztóczi[1], Thomas Forro[1], Thomas Klausberger [1*]

[1]Divison of Cognitive Neurobiology, Center for Brain Research, Divison of Cognitive Neurobiology, 1090 Vienna, Austria

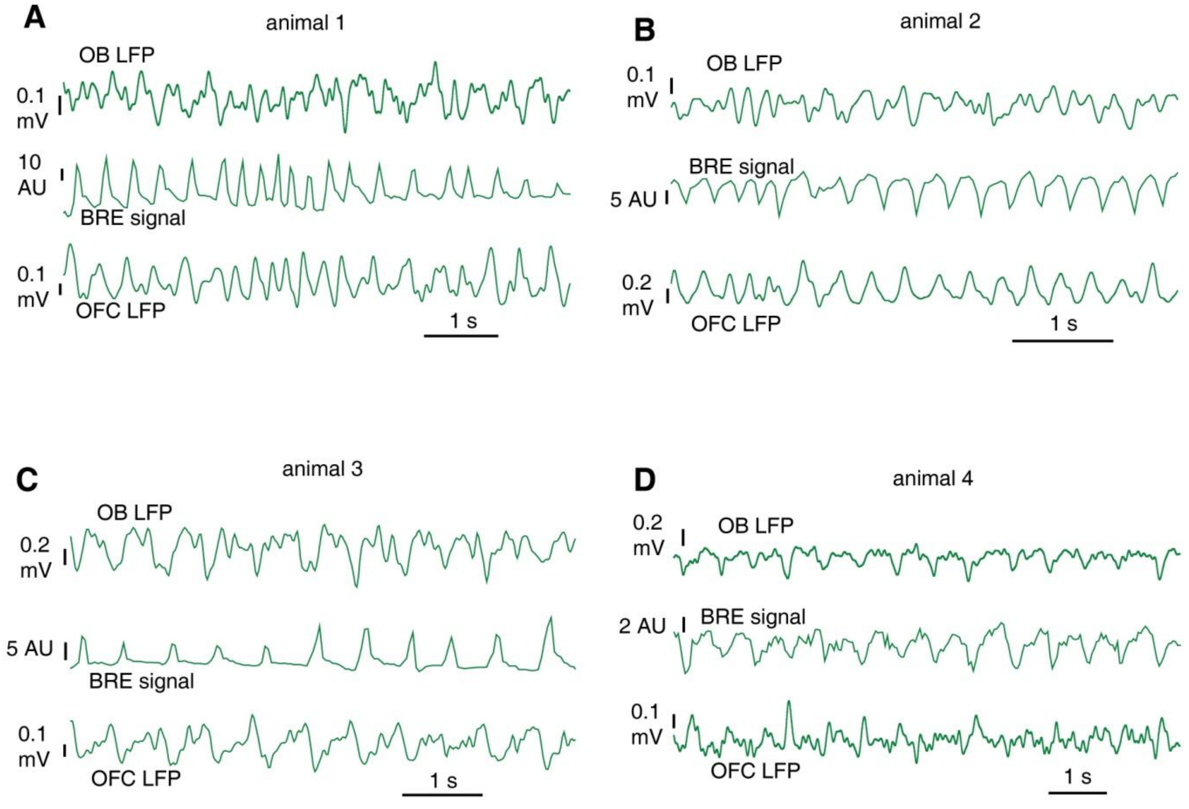

Supplementary Figure 1. Slow oscillatory components in the LFP of olfactory bulb and orbitofrontal cortex, coupling to the breathing signal (**A-D**) LFP and breathing signal traces from all four experimental animals from which the silicon probe recorded orbitofrontal units were gathered. For all panels top row is always OB LFP (12Hz low-pass filtered), middle row is breathing signal, bottom row is OFC LFP. The source of the breathing signal for panel B and D was an appropriately selected ROI at the back contour of the animal on the video, whereas for A and C an abdominal contour region.

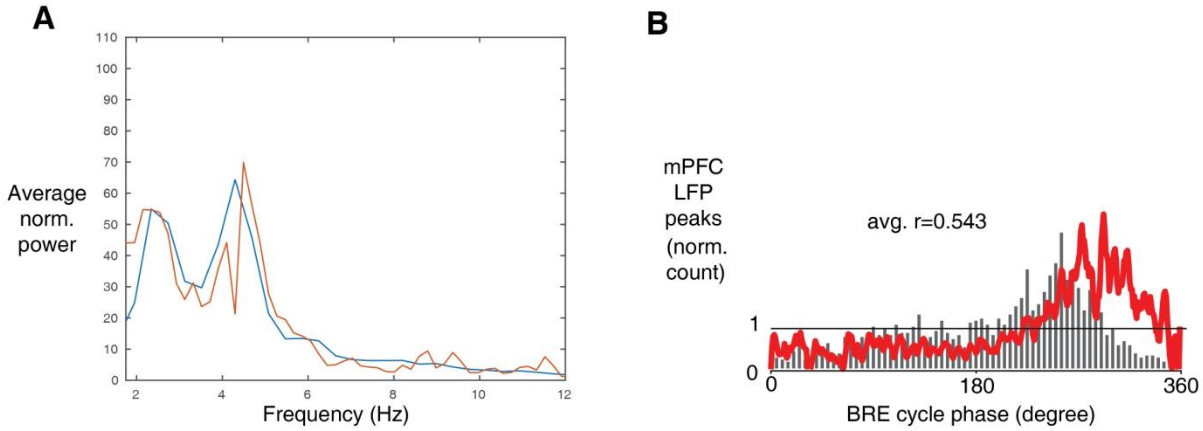

Supplementary Figure 2. Breathing coherent slow oscillatory component in the mPFC LFP (**A**) Average power spectrums from the breathing signal (red) and from the medial prefrontal LFP (blue, from 2 mice); note the strong overlap. (**B**) Phase histogram showing the distribution of mPFC slow LFP peaks over the breathing cycle phases, from a single session (gray bars), and average phase histogram from 2 sessions, from two different animals (red line). The slow oscillatory cycles in the LFP were significantly coupled to the breathing cycles (Rayleigh test), average r value was 0.543.
